# The impact of plant-based diets on female bone mineral density: Evidence based on seventeen studies

**DOI:** 10.1097/MD.0000000000027480

**Published:** 2021-11-19

**Authors:** Xingfa Ma, Haoyin Tan, Mingjun Hu, Shengcai He, Lijuan Zou, Huashan Pan

**Affiliations:** a School of Sports and Health, Guangzhou University of Chinese Medicine, Guangzhou, China; b ZhuHai Health School, ZhuHai, China; c GuangDong Food and Drug Vocational College Science and Technology Division, Guangzhou, China.

**Keywords:** bone health, bone mineral density, meta-analysis, plant-based diets

## Abstract

**Background:**

An increase in awareness of plant-based diets has brought forth numerous studies on bone mineral density (BMD). The present systematic review and meta-analysis was designed to compare the effect between plant-based diets and omnivores on female BMD.

**Methods:**

We searched the Cochrane Library, PubMed, EMBASE, and Web of Science and up to July 1, 2020. Mean difference (MD) with its 95% confidence interval (CI) was estimated to compare the outcomes of the groups. We compared BMD at the lumbar spine, femoral neck and whole body respectively between plant-based diets and omnivores. In addition, we performed subgroup analyses according to different clinical characteristics for further exploration. Two reviewers assessed trial quality and extracted data independently. All statistical analyses were performed using standard statistical procedures provided in Review Manager 5.2.

**Results:**

A total of 17 cross-sectional studies including 13,888 patients were identified for the present meta-analysis. Our pooled result indicated that population with plant-based diets had lower BMD than omnivores at the lumbar spine (MD −0.03; 95% CI −0.04 to −0.02; *P* < .0001), femoral neck (MD −0.04; 95% CI −0.05 to −0.03; *P* < .00001) and whole body (MD −0.04; 95% CI −0.06 to −0.01; *P* = .01), respectively. Further exploration indicated that especially females with plant-based diets experienced significantly lower BMD at lumbar spine (MD −0.03; 95% CI −0.04 to −0.02; 3173 pts), femoral neck (MD −0.04; 95% CI −0.05 to −0.03; 10,656 pts) and whole body (MD −0.05; 95% CI −0.10 to −0.00; *P* = .04). In addition, we performed subgroup analyses and found lower BMD at lumbar spine and femoral neck in both vegetarians and vegans.

**Conclusions:**

The present meta-analysis indicated that plant-based diets may be correlated with lower BMD of women when compared with omnivore population. However, this does not diminish the fact that a plant-based diet can be a harmful option to the overall bone health of population and more prospective researches are needed to clear the impact of plant-based diets on bone health.

## 1 Introduction

Vegetarian diets continue to gain popularity given the widespread concerns about environmental sustainability and the belief that a healthier diet prevents chronic disease development.^[1–3]^ Also, large populations adhere to vegetarian or vegan diets for cultural or religious reasons.^[4]^ Many studies on the effects of such diets on bone health have appeared.^[5–7]^ In the USA, osteoporosis was associated with low bone mineral density (BMD) and was evident in 5% of men and 25% of women aged >65 years.^[8,9]^ Osteoporosis is responsible for >8.9 million fractures annually worldwide, increasing morbidity and mortality and imposing large economic burdens.^[8,9]^ Therefore, identifying and addressing factors associated with poor bone health is a public health imperative. Meta-analyses have suggested that vegetarians and vegans exhibit lower BMDs and a higher risk of fractures than omnivores.^[10,11]^

Consistent with other reports,^[10,11]^ adult respondents to the 2007 to 2010 NHANES surveys who self-identified as vegetarians (including vegans) exhibited significantly lower BMDs than nonvegetarians.^[12]^ However, further analysis indicated that the lower BMD at the hip (the femur and femoral neck) was attributable to a smaller body size when anthropometric (body mass index [BMI] and waist circumference) corrections were applied.^[13]^ This was not the case for the vegetarian lumbar spine BMD, which remained significantly different from that of nonvegetarians after adjustment for anthropometric variables. Also, and importantly, the small lumbar spine BMD difference was of minimal clinical significance, being only <0.05 g/cm^2^ (< 3%).^[13,14]^

In addition, several reviews have examined the current literatures regarding the evidence of the association between plant-based diets and low BMD.^[15–17]^ However, their conclusions still remained inconsistency. Hsu (2020)^[15]^ considered that insufficient calcium and/or vitamin D intake was detrimental to bone metabolism and bone health and, with planning and a balanced diet, vegans can obtain healthy levels of calcium and vitamin. In contrast, Iguacel (2019)^[11]^ indicated that vegetarian and vegan diets should be planned to avoid negative consequences on bone health, because compared with omnivores, vegetarians, and vegans had lower BMD at the femoral neck and lumbar spine and vegans also had higher fracture rates. Further, the influence of plant-based diets in different sites like lumbar spine, femoral neck, and whole body are still unclear. Thus, we designed this systematic review and meta-analysis focused on the effect of plant-based diets on different sites of body BMD comprehensively. In addition, we conducted subgroup analysis in order to explore the factors of BMD including diets, age, detection instruments, gender, and population ethnicity.

## 2 Methods and materials

### 2.1 Criteria for considering studies

We included studies if they meet the following criteria: an observational study (prospective or retrospective) comparing plant-based and omnivorous diets; a human study; and, BMD measurement via imaging. The exclusion criteria were: inclusion of subjects with bone metabolic diseases; an experimental trial involving animals, or any other nonhuman study; an abstract, letter, editorial, expert opinion, review, or case report; and, a study lacking sufficient data or that did not meet any inclusion criterion.

The present study was approved by the Ethics Committee of The First Affiliated Hospital of Guangzhou University of Chinese Medicine.

### 2.2 Search strategy

We searched the Cochrane Library, PubMed, EMBASE, and Web of Science up to July 1, 2020. Our strategy was based on combinations of medical subject headings and the keywords:“plant”, “Vegan”, “Vegans”, “Vegetarians”, “Vegetarian”, “Bone”, “bone mineral density”, and “Osteoporosis”. Two assessors independently screened the titles and abstracts of each study. Once relevant studies became certain, the full texts were obtained for further evaluation. Other related references we read were also searched online for full texts and assessment, once any of them meet our including criteria, they will also be included in this meta-analysis.

### 2.3 Quality assessment and data extraction

Two reviewers assessed the quality of each included study using the previously validated 11-item checklist which was recommended by Agency for Healthcare Research and Quality (AHRQ).^[18]^ An item would be scored “0” if it was answered “NO” or “UNCLEAR”; if it was answered “YES”, then the item scored “1”. Article quality was assessed as follows: low quality = 0 to 3; moderate quality = 4 to 7; high quality = 8 to 11. In addition, the risk of bias for each study and the risk of bias across all studies were evaluated and shown with figures generated by RevMan 5.2 software (Copenhagen, Denmark).^[19]^

Data for the comparative analysis of plant-based diets versus omnivores for BMD were extracted independently by 2 reviewers, and disagreement was resolved through discussion. The extracted contents, including first authors, published years, country, sample size, interventions, baseline, and age of each study, were displayed using a standardized form. Data collected were input into RevMan 5.2 software for analysis.^[19]^

### 2.4 Statistical analysis

The data of comparable outcomes between plant-based diets and omnivores were combined-analyzed, using the standard statistical procedures provided in RevMan 5.2.^[19]^ We compared BMD at the lumbar spine, femoral neck, and whole body respectively between plant-based diets and omnivores. In addition, we performed subgroup analyses according to different clinical characteristics for further exploration. Mean difference (MD) with its 95% confidence interval (CI) was estimated to compare the outcomes of the groups. The heterogeneity between studies was evaluated by the chi-square-based Q statistical test,^[20]^ with *P*_*h*_ value, and *I*^*2*^ statistic, ranging from 0% to 100%, to quantify the effect of heterogeneity. *P*_*h*_ ≤ 0.10 was deemed to represent significant heterogeneity,^[21]^ and pooled estimates were estimated using a random-effect model (the DerSimonian and Laird^[22]^ method). On the contrary, if statistical study heterogeneity was not observed (*P*_*h*_ > 0.10), a fixed effects model (the Mantel–Haenszel^[23]^ method) was used. The effects of outcome measures were considered to be statistically significant if pooled MDs with 95% CI did not overlap with 0.

This work has been reported in line with preferred reporting items for systematic reviews and meta-analyses^[24]^ and Assessing the methodological quality of systematic reviews Guidelines.^[25]^ Our research was registered in “Research registry”.

## 3 Results

### 3.1 Included studies, study characteristics, and quality assessment

**Figure 1:**
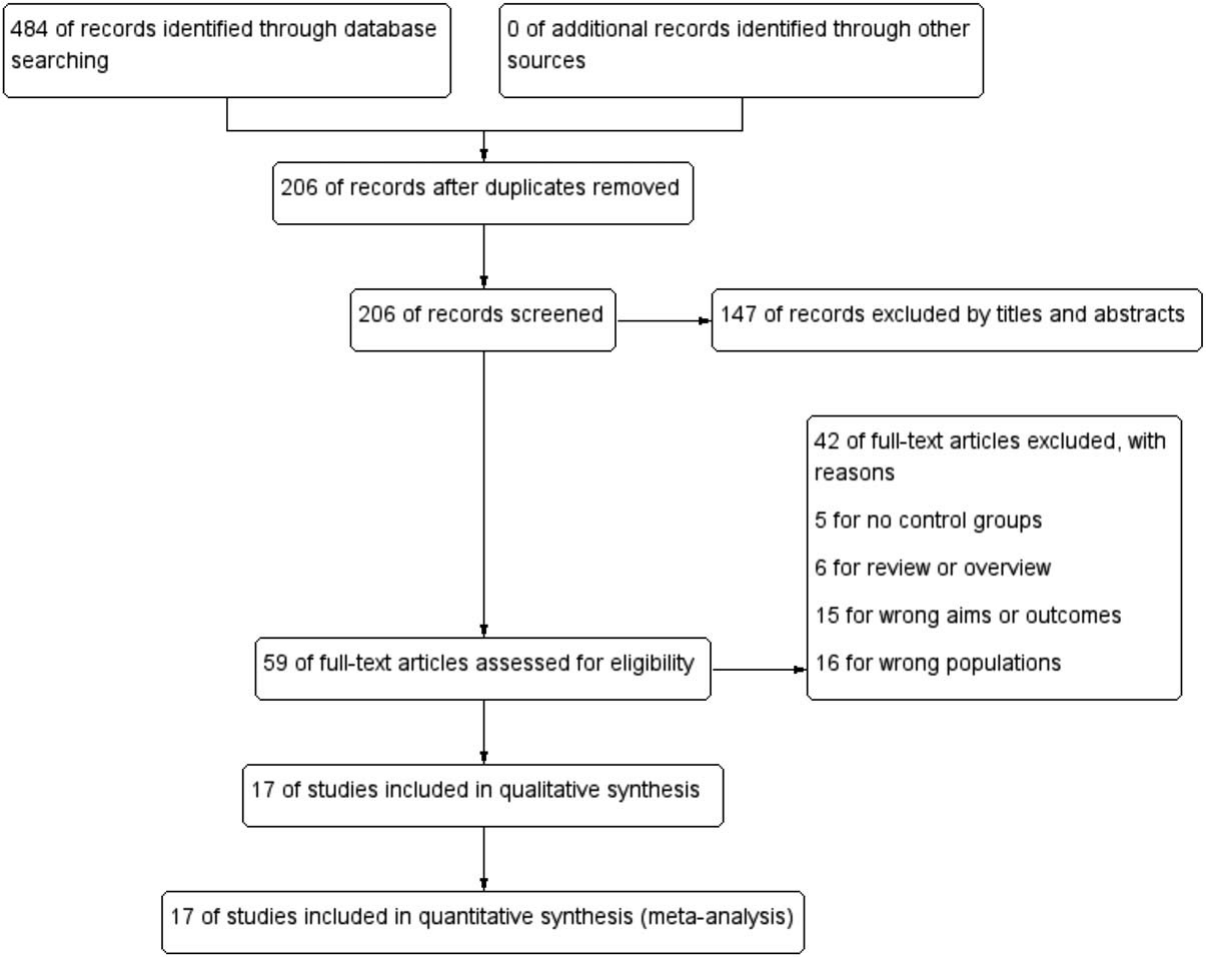
Flow diagram of literature search and selection of included studies for meta-analysis.

At the beginning of the search, a total of 484 records of citations were obtained; 206 of records were obtained further after duplicates were removed. Via screening the titles and abstracts, 147 studies were excluded preliminarily and then 59 studies were chosen to get full texts for further evaluation. After reading the full texts of the 59 studies, 42 studies were excluded further (16 studies for wrong populations, 6 for reviews, 5 for no controls, and 15 for wrong aims). Finally, 17 cross-sectional studies^[26–41]^ including 13,888 subjects were reviewed and meta-analyzed. The detailed search process and summary of studies were shown in the study flow diagram (Fig. 1). Of these studies, 14 studied BMD at lumbar spine, 9 studied BMD at femoral neck, and 5 studied BMD at whole body. Five studies came from China and 4 from USA. Nine study populations were Caucasian and 7 Asian. Twelve studies measured BMD via dual-energy X-ray absorptiometry (DXA) and 4 via dual-photon absorptiometry (DPA). The other characteristics of each study were shown in Table 1.

**Table 1:** The characteristics of included studies for this meta-analysis.

		Omnivores			Plant-based diets						
Study	Country	No.	Age (M ± SD)	BMD	No.	Age (M ± SD)	BMD	BMD site	Gender	Ethnicity	Instruments
Barr et al (1998)	Canada	22	27.9 ± 5.9	1.216 ± 0.132	15 8	25.8 ± 4.7 28.0 ± 3.2	1.145 ± 0.117 1.153 ± 0.100	LS	Female	Caucasian	DXA
Chiu et al (1997)	Taiwan, China	187	59.5 ± 8.0	0.990 ± 0.170 0.750 ± 0.130	71	64.0 ± 11.5	0.940 ± 0.190 0.690 ± 0.110	LS FN	Female	Asian	DPA
Fontana et al (2005)	USA	7	53.2 ± 4.1	1.030 ± 0.140	7	56.5 ± 13.1	0.850 ± 0.080	LS	Female	Caucasian	DXA
				0.750 ± 0.080			0.630 ± 0.100	FN			
				1.100 ± 0.100			0.990 ± 0.006	WB			
Ho-Pham et al (2009)	Vietnam	105	62.0 ± 10.0	0.770 ± 0.140	105	62.0 ± 10.0	0.740 ± 0.140	LS	Female	Asian	DXA
				0.630 ± 0.110			0.620 ± 0.110	FN			
				0.900 ± 0.120			0.880 ± 0.110	WB			
Karavasiloglou et al (2020)	Germany	9209	47.0 ± 0.4	0.94 ± 0.110 0.80 ± 0.120	207	44.4 ± 1.3	0.90 ± 0.120 0.75 ± 0.130	LS FN	Male female	Multi-race/ethnicity	DXA
Kim et al (2007)	Korea	76	60.8 ± 6.7	0.809 ± 0.158	76	60.7 ± 6.9	0.806 ± 0.140	LS	Female	Asian	DXA
				0.711 ± 0.112			0.684 ± 0.144	FN			
Knurick et al (2015)	USA	27	27.2 ± 6.7	1.180 ± 0.110	27	31.1 ± 9.1	1.120 ± 0.100	WB	Female Male	Caucasian	DXA
					28	33.9 ± 8.6	1.130 ± 0.110				
Krivoskova et al (2010)	Slovakia	131	40.8 ± 19.8	1.102 ± 0.159	141	41.9 ± 19.7	1.085 ± 0.192	LS	Female	Caucasian	DXA
				0.941 ± 0.136			0.918 ± 0.142	FN			
Kaur (2013)	India	46	45.0 ± 80.0	0.888 ± 0.140	204	45.0 ± 80.0	0.872 ± 0.118	LS	Female	Caucasian	DXA
Lloyd et al (1991)	USA	36	36.1 ± 0.4	1.006 ± 0.120	23	35 ± 0.7	1.020 ± 0.096	LS	Female	Caucasian	DPA
Lau et al (1998)	Hong Kong, China	109	77.0 ± 3.8	0.720 ± 0.140	40	79.9 ± 5.4 78.2 ± 4.9	0.680 ± 0.110 0.720 ± 0.150	LS	Female	Asian	DPA
				0.530 ± 0.082	36		0.480 ± 0.080 0.500 ± 0.080	FN			
Outila et al (2000)	Finland	16	34.0 ± 7.0	1.177 ± 0.099	6	33.0 ± 9.0	1.138 ± 0.060 1.034 ± 0.174	LS	Female	Caucasian	DXA
				0.999 ± 0.138		37.0 ± 7.0	0.961 ± 0.059 0.843 ± 0.116	FN			
Siani et al (2003)	Italy	10	38.4 ± 7.8	1.190 ± 0.110	20	34.8 ± 15.1	1.190 ± 0.070	WB	Female Male	Caucasian	DXA
Tesar et al (1992)	USA	28	62.9 ± 5.6	1.066 ± 0.155	28	62.9 ± 5.1	1.079 ± 0.203	LS	Female	Caucasian	DPA
Wang et al (2008)	Taiwan, China	529	21.0 ± 89.0	0.968 ± 0.183	489	21.0 ± 89.0	0.953 ± 0.179	LS	Female	Asian	DXA
		463		0.829 ± 0.142	383		0.813 ± 0.127	FN	Male		
Xie et al (2019)	China	246	32.1 ± 6.5	1.519 ± 0.331	246	32.7 ± 6.5	1.519 ± 0.310	WB	Female Male	Asian	DPA
Ying-Ming and Liu (2010)	China	302	50.0 ± 70.0	0.837 ± 0.140	173	50.0 ± 70.0	0.795 ± 0.140	LS	Female	Asian	DXA

BMD = bone mineral density, DPA = dual-photon absorptiometry, DXA = dual-energy X-ray absorptiometry, FN = femoral neck, LS = lumbar spine, M = mean, NR = not report, SD = standard deviation, WB = whole body.

**Figure 2:**
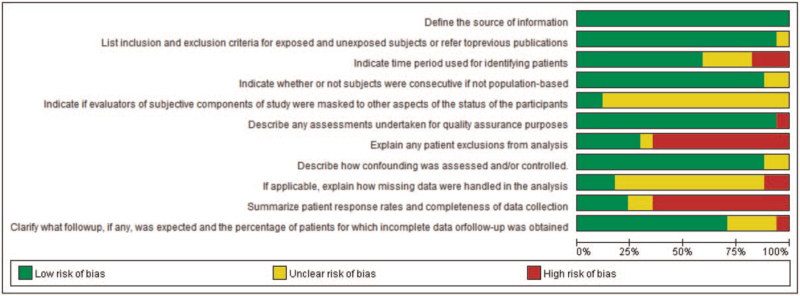
Risk of bias graph: review authors’ judgements about each risk of bias item presented as percentages across all included studies.

**Figure 3:**
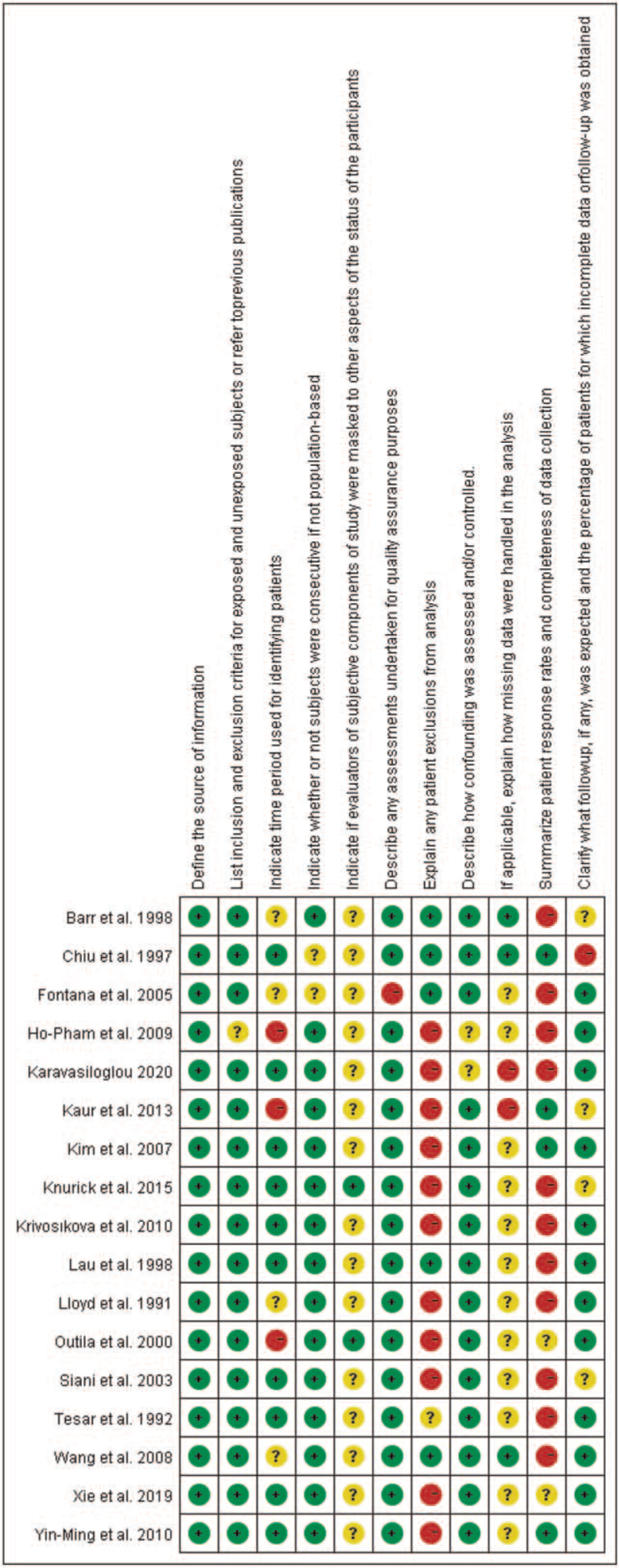
Risk of bias summary: review authors’ judgements about each risk of bias item for each included study.

According to our definitions, there was no low quality studies included in this analysis. Eleven studies were assessed as moderate quality (64.7%) and 6 studies were evaluated as high quality. Additionally, risk-of-bias graphs were generated to further identify the risk of bias of the including studies. The risk of bias for each RCT was presented as percentages across all included studies, and the risk-of-bias item for each included study was displayed (Figs. 2 and 3). The risk-of-bias graphs indicated generally low risk of bias in the AHRQ item of “Define the source of information (survey, record review)”, “List inclusion and exclusion criteria for exposed and unexposed subjects (cases and controls) or refer to previous publications”, “Indicate whether or not subjects were consecutive if not population-based”, “Describe any assessments undertaken for quality assurance purposes”, and “Describe how confounding was assessed and/or controlled”. High risk of bias was mainly observed in item of “Explain any patient exclusions from analysis” and “Summarize patient response rates and completeness of data collection”. An unclear risk of bias was mainly observed in “Indicate if evaluators of subjective components of study were masked to other aspects of the status of the participants” and “If applicable, explain how missing data were handled in the analysis”. Table S1, Supplemental Digital Content.

### 3.2 Effect of plant-based diets on BMD at the lumbar spine

**Figure 4:**
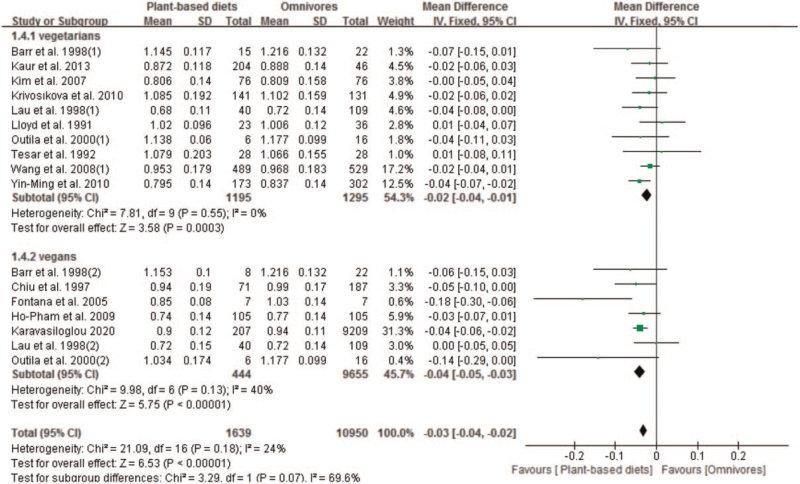
Forest plot of effect of vegetarian diets on bone mineral density at the lumbar spine. CI = confidence interval, SD = standard deviation.

A total of 14 studies with 17 sets of data compared the effect of plant-based diets and omnivores on lumbar spine BMD. Our results showed that plant-based diets population experienced lower BMD than omnivores at the lumbar spine, with a pooled MD of −0.04 (95% CI −0.05 to −0.03; *P* < .00001) (Fig. 4). The pooled analysis was performed using a fixed-effect model because no significant in-study heterogeneity was observed (*P* < .18 and *I*^*2*^ = 24%). Subgroup analysis found significantly lower BMD in both vegetarians (MD −0.02; 95% CI −0.04 to −0.01; 2490 pts) and vegans patients (MD −0.04; 95% CI −0.05 to −0.03; 10099 pts), respectively (Fig. 4).

In addition, we further conducted subgroup analysis with the following categories: year, age, study quality, instruments, ethnicity, sample of size, and gender. As showing in Table 2, significant reduction of BMD at the lumbar spine was found in the following subgroups: before 2000 (MD −0.03; 95% CI −0.05 to −0.00; *P* = .02) and after 2000 (MD −0.03; 95% CI −0.04 to −0.02; *P* < .0001); mean age ≥ 50 year (MD −0.03; 95% CI −0.04 to −0.02; *P* < .0001); high study quality (MD −0.04; 95% CI −0.05 to −0.02; *P* < .0001); DXA (MD −0.03; 95% CI −0.04 to −0.02; *P* < .0001); Caucasian (MD −0.03; 95% CI −0.05 to −0.01; *P* = .01) and Asian (MD −0.03; 95% CI −0.04 to −0.01; *P* < .0001); sample size ≤ 100 pts (MD −0.05; 95% CI −0.01 to 0.00; *P* = .03) and >100 pts (MD −0.03; 95% CI −0.04 to −0.02; *P* < .0001); female (MD −0.03; 95% CI −0.04 to −0.02; *P* < .0001), and male/female (MD −0.04; 95% CI −0.06 to −0.02; *P* < .0001). However, no significant difference was found in subgroup of mean age <50 year (MD −0.03; 95% CI −0.06 to 0.00; *P* = .08), moderate quality studies (MD −0.02; 95% CI −0.04 to 0.00; *P* = .08), and DPA (MD −0.02; 95% CI −0.04 to 0.00; *P* = .07), respectively (Table 2).

**Table 2:** Subgroup analyses of the effect of plant-based diets on bone mineral density at the lumbar spine.

		Pooled results			Heterogeneity	
Subgroups	No. of study/pts	MD	95% CI	*P*-value	*I* ^2^	Analytical effect model
Year						
Before 2000	7/738	−0.03	−0.05 to −0.00	.02	10%	Fixed effect model
After 2000	10/11,851	−0.03	−0.04 to −0.02	<.0001	37%	Fixed effect model
Mean age						
<50 yr	6/185	−0.03	−0.06 to 0.00	.08	29%	Fixed effect model
≥50 yr	11/12,147	−0.03	−0.04 to −0.02	<.0001	31%	Fixed effect model
Quality						
Moderate	7/2016	−0.02	−0.04 to 0.00	.08	51%	Random-effect model
High	10/10,085	−0.04	−0.05 to −0.02	<.0001	0%	Fixed effect model
Instruments						
DXA	11/11,646	−0.03	−0.04 to −0.02	<.0001	34%	Fixed effect model
DPA	6/943	−0.02	−0.04 to 0.00	.07	0%	Fixed effect model
Ethnicity						
Caucasian	9/762	−0.03	−0.05 to −0.01	.01	42%	Fixed effect model
Asian	7/2411	−0.03	−0.04 to −0.01	<.0001	0%	Fixed effect model
Sample size						
≤100 pts	7/240	−0.05	−0.01 to 0.00	.03	53%	Random-effect model
>100 pts	10/12,349	−0.03	−0.04 to −0.02	<.0001	0%	Fixed effect model
Gender						
Female	9/3173	−0.03	−0.04 to −0.02	<.0001	22%	Fixed effect model
Male/female	1/9416	−0.04	−0.06 to −0.02	<.0001	–	–

CI = confidence intervals, DPA = dual-photon absorptiometry, DXA = dual-energy X-ray absorptiometry, MD = mean difference.

### 3.3 Effect of plant-based diets on BMD at the femoral neck

**Figure 5:**
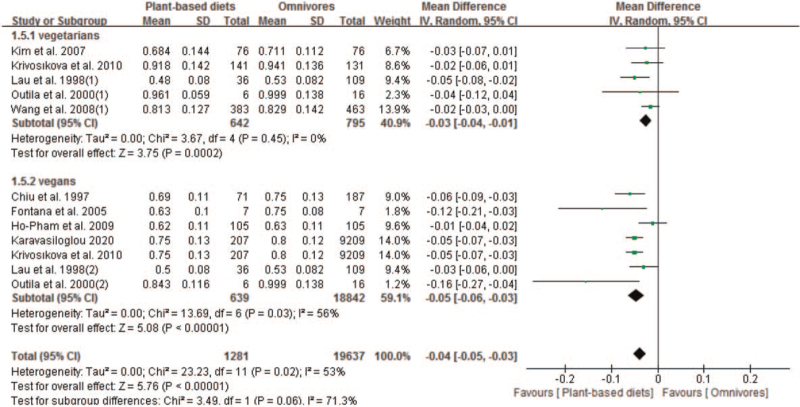
Forest plot of effect of vegetarian diets on bone mineral density at the femoral neck. CI = confidence interval, SD = standard deviation.

We included 10 studies with 12 sets of data that compared the effect of plant-based diets and omnivores on femoral neck BMD. Our results showed that plant-based diets population experienced lower BMD than omnivores at the femoral neck, with a pooled MD of −0.04 (95% CI −0.05 to −0.03; 20,918 pts). The pooled analysis was performed using a random-effect model because a significant in-study heterogeneity was observed (*P* = .02 and *I*^*2*^ = 53%) (Fig. 5). Subgroup analysis found significantly lower BMD in both vegetarians (MD −0.03; 95% CI −0.04 to −0.01; *P* = .0002) and vegans patients (MD −0.05; 95% CI −0.06 to −0.03; *P* < .00001), respectively (Fig. 5).

We also conducted subgroup analysis for the effect of plant-based diets on BMD at the femoral neck. As showing in Table 3, significant reduction of BMD at the femoral neck was found in both before 2000 (MD −0.05; 95% CI −0.06 to −0.03; *P* < .0001) and after 2000 (MD −0.03; 95% CI −0.05 to −0.01; *P* = .0003). In subgroup analysis of population ethnicity, significant reduction of femoral neck BMD was observed in both Caucasians (MD −0.05; 95% CI −0.06 to −0.03), and Asians (MD −0.03; 95% CI −0.05 to −0.01). In subgroup analysis of gender, significant reduction of femoral neck BMD was observed in female population (MD −0.04; 95% CI −0.05 to −0.03), but not male population (MD −0.02; 95% CI −0.03 to 0.00). Significant reduction was also found in both high AHRQ quality (MD −0.04; 95% CI −0.05 to −0.03) and moderate AHRQ quality (MD −0.02; 95% CI −0.04 to −0.00). In subgroup analysis of sample size, significant reduction was observed in both sample size <100 patients (MD −0.09; 95% CI −0.15 to −0.04) and sample size ≥100 patients (MD −0.03; 95% CI −0.04 to −0.02). In subgroup analysis of mean age, significant reduction was observed in both age <50 years (MD −0.07; 95% CI −0.13 to −0.01; *P* = .02) and ≥50 year (MD −0.03; 95% CI −0.05 to −0.02; *P* < .0001); in subgroup analysis of instruments, significant reduction was observed in both DXA (MD −0.03; 95% CI −0.05 to −0.01; *P* = .0009) and DPA (MD −0.05; 95% CI −0.06 to −0.03; *P* < .0001) (Table 3).

**Table 3:** Subgroup analyses of the effect of plant-based diets on bone mineral density at the femoral neck.

		Pooled results			Heterogeneity	
Subgroups	No. of study/pts	MD	95% CI	*P*-value	*I* ^2^	Analytical effect model
Year						
Before 2000	3/548	−0.05	−0.06 to −0.03	<.0001	0%	Fixed effect model
After 2000	8/10,954	−0.03	−0.05 to −0.01	.0003	59%	Random-effect model
Mean age						
<50 yr	4/330	−0.07	−0.13 to −0.01	.02	61%	Random-effect model
≥50 yr	7/11,172	−0.03	−0.05 to −0.02	<.0001	55%	Random-effect model
Instruments						
DXA	8/10,954	−0.03	−0.05 to −0.01	.0009	59%	Random-effect model
DPA	3/548	−0.05	−0.06 to −0.03	<.0001	0%	Fixed effect model
Ethnicity						
Caucasians	5/9746	−0.05	−0.06 to −0.03	<.0001	49%	Fixed effect model
Asians	5/777	−0.03	−0.05 to −0.01	<.0001	45%	Fixed effect model
Gender						
Female	10/10,656	−0.04	−0.05 to −0.03	<.0001	42%	Fixed effect model
Male	1/846	−0.02	−0.03 to 0.00	.08	–	–
Male / female	1/9416	−0.05	−0.07 to −0.03	<.0001	–	–
Quality						
Moderate	4/1284	−0.02	−0.04 to −0.01	.004	34%	Fixed effect model
High	7/10,218	−0.04	−0.05 to −0.03	<.0001	46%	Fixed effect model
Sample size						
≤100 pts	3/58	−0.09	−0.15 to −0.04	.001	37%	Fixed effect model
>100 pts	8/11,444	−0.03	−0.04 to −0.02	<.0001	46%	Fixed effect model

CI = confidence intervals, MD = mean difference.

### 3.4 Effect of plant-based diets on BMD at the whole body

**Figure 6:**
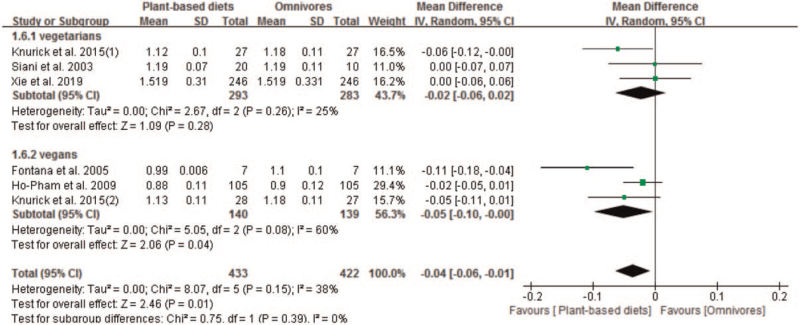
Forest plot of effect of vegetarian diets on bone mineral density at the whole body. CI = confidence interval, SD = standard deviation.

We identified 5 studies with 6 sets of data that compared the effect of plant-based diets and omnivores on BMD at the whole body. The BMD at the whole body in plant-based diets group was lower than omnivores group, with a MD of −0.04 (95% CI −0.06 to −0.01; *P* = .01). The pooled analysis was performed using a random effect model because significant heterogeneity was observed (*P* = .08 and *I*^*2*^ = 60%) in subgroup (Fig. 6). Subgroup analysis indicated significant reduction of BMD at the whole body in vegans (MD −0.05; 95% CI −0.10 to −0.00; *P* = .04). However, no significant reduction of BMD at the whole body in vegetarians was observed, with a pooled MD of −0.02 (95% CI −0.06 to 0.02; *P* = .28) (Fig. 6).

Subgroup analysis indicated significant reduction of BMD at the whole body in plant-based diets group in subgroups of publish year after 2010 (MD −0.04; 95% CI −0.07 to −0.00; *P* = .03), Mean age ≥ 50 years (MD −0.03; 95% CI −0.05 to −0.01; *P* = .02), high AHRQ quality (MD −0.03; 95% CI −0.05 to −0.01; *P* = .02), DXA (MD −0.04; 95% CI −0.06 to −0.01; *P* = .001), Caucasians (MD −0.06; 95% CI −0.09 to −0.02; *P* = .0007), sample size ≤100 pts (MD −0.06; 95% CI −0.09 to −0.02; *P* = .0007) and female (MD −0.05; 95% CI −0.10 to −0.00; *P* = .04). No significant difference of BMD at the whole body between plant-based diets and omnivores was observed in subgroups of publish year before 2010, mean age <50 years, DPA, Asians, moderate AHRQ quality, male/female and sample size >100 pts, respectively (Table 4).

**Table 4:** Subgroup analyses of the effect of plant-based diets on bone mineral density at the whole body.

		Pooled results			Heterogeneity	
Subgroups	No. of study/pts	MD	95% CI	*P*-value	*I* ^2^	Analytical effect model
Year						
Before 2010	3/254	−0.04	−0.10 to 0.02	. 17	63%	Random-effect model
After 2010	3/601	−0.04	−0.07 to −0.00	.03	19%	Fixed effect model
Mean age						
<50 yr	2/44	−0.06	−0.16 to 0.05	.32	76%	Random-effect model
≥50 yr	4/811	−0.03	−0.05 to −0.01	.02	0%	Fixed effect model
Quality						
Moderate	2/44	−0.06	−0.16 to 0.05	.32	76%	Random-effect model
High	4/811	−0.03	−0.05 to −0.01	.02	0%	Fixed effect model
Instruments						
DXA	5/363	−0.04	−0.06 to −0.01	.001	40%	Fixed effect model
DPA	1/492	0.00	−0.06 to 0.06	1.00	–	–
Ethnicity						
Caucasians	4/153	−0.06	−0.09 to −0.02	.0007	29%	Fixed effect model
Asians	2/702	−0.02	−0.04 to 0.01	.27	0%	Fixed effect model
Sample size						
≤100 pts	4/153	−0.06	−0.09 to −0.02	.0007	29%	Fixed effect model
>100 pts	2/702	−0.02	−0.04 to 0.01	.27	0%	Fixed effect model
Gender						
Female	3/279	−0.05	−0.10 to −0.00	.04	60%	Random-effect model
Male/female	3/576	−0.02	−0.06 to 0.01	.19	25%	Fixed effect model

CI = confidence intervals, MD = mean difference.

### 3.5 Publication bias

**Figure 7:**
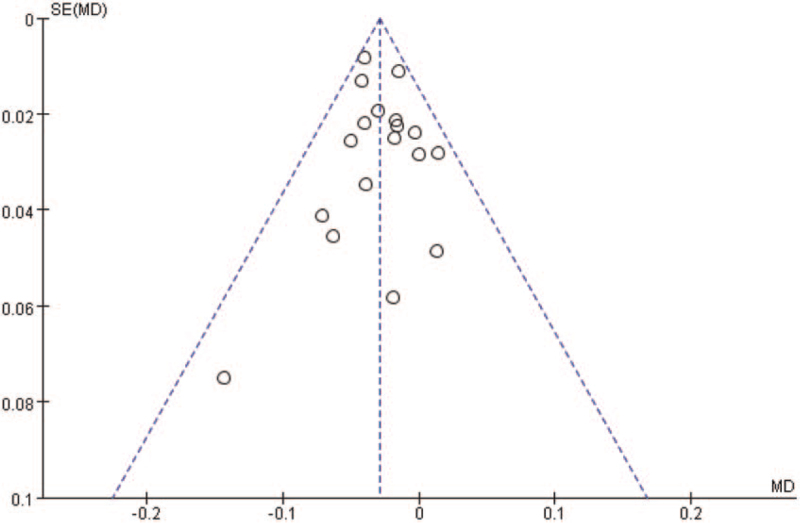
Begg funnel plot for detecting publication bias. MD = mean difference.

Begg funnel plot was generated to assess publication bias in the included studies. As shown in Figure 7, the plots displayed no obvious asymmetry and showed no clear evidence of publication.

## 4 Discussion

Nutrition and food intake have long been key components of health. Diets abstaining from animal products have existed for millennia for a diversity of reasons ranging from religious to ethical concerns.^[15]^ In recent times, plant-based diets have dramatically risen in public awareness, particularly as meat and dairy products are linked to higher environmental impact and drivers of human-induced climate change.^[42]^

Current research indicates a possible link between decreased calcium/vitamin D levels in the body and low BMD. Low BMD is used as one of the main diagnostic criteria for osteoporosis.^[43]^ The possible connection between a vegan lifestyle, low BMD and the development of osteoporosis has important societal as well as medical implications. Low BMD and the development of osteoporosis often lead to fractures – hip, spine and wrist fractures being the most common.^[44]^ Incidence of hip fractures results in costly medical procedures that tax the health-care system and increased morbidity and mortality rates.^[44]^

However, because the reason for osteoporosis and fracture was multifactorial, many current researches failed to clear the effect of plant-based diets on bone health. Low BMD has been demonstrated correlated to osteoporosis and fractures. Thus, we conducted this meta-analysis and systematic review aiming to comprehensively compare the effect between plant-based diets and omnivores on BMD. We found that population with plant-based diets had lower BMD than omnivores at the lumbar spine (MD −0.04; 95% CI −0.06 to −0.02), femoral neck (MD −0.04; 95% CI −0.05 to −0.02) and whole body (MD −0.03; 95% CI −0.06 to −0.01), respectively. In addition, we performed subgroup analyses and found lower BMD at lumbar spine, femoral neck, and whole body in both vegetarians and vegans. Previous studies have suggested that vegetarians may have higher BMD and bone mineral content than omnivores.^[34,45–47]^ However, recent studies have not found any positive impact of vegetarian diets on bone health and some of them have found a negative impact.^[28,32,33,35]^ The present results were in line with a previous meta-analysis and found lower BMD at the lumbar spine and the femoral neck for vegetarians compared with omnivores.^[10,11]^ Ho-Pham et al^[10]^ (2009) concluded that vegetarians and vegans had approximately 4% lower BMD at the lumbar spine and femoral neck than omnivores. In addition, our subgroup analysis found both vegetarians and vegans lead to lower BMD at the lumbar spine, the femoral neck, and whole body. Both Caucasians and Asians population were found having lower BMD at the lumbar spine and femoral neck. The lower BMD at the lumbar spine was found in both mean age <50 year and ≥ 50 year. For BMD at the femoral neck, we found that the significant reduction was observed in female participants but not in male participants.

It is worth noting that lifestyle factors may have influenced the associations between diet and BMD. Vegetarians and vegans tend to show healthier behaviors, such as higher levels of physical activity, lower smoking rates, and lower alcohol and caffeine intakes, than omnivores.^[48]^ Particularly, in the studies included in this systematic review and meta-analyses, vegans and vegetarians generally reported higher levels of physical activity, lower smoking rates, lower alcohol and caffeine intakes, lower BMI, and lower energy and calcium intakes than omnivores, although some of these group differences were not statistically significant in several studies.^[33,36]^

In the relationship between vegetarian/vegan diets and bone health, it is important to consider the possible effect that overall dietary quality can have. Among the studies included in the present systematic review and meta-analysis, only 1 study considered overall dietary quality.^[31]^ In this study, diet quality was superior for individuals adhering to a vegan diet as compared with the other diet groups, and there were no differences in BMD among vegans, vegetarians, and omnivores, which suggested that a high-quality vegan/vegetarian diets would look similar to that of an omnivore in relation to bone health. Our results indicated significantly lower BMD in female with plant-based diets, which may be mainly related to hormonal changes in postmenopausal women. Long-term female vegetarians may need effective nutritional supplements (particularly calcium and Vitamin D) to increase BMD levels and reduce the risk of osteoporosis.^[49]^

The present study had several limitations. First, most of the studies in the present meta-analysis included only women, and hence results are mainly applicable to this population. Second, investigations included a very heterogeneous population (ie, some of the studies focused on Buddhist nuns or religious followers of Buddhism, whereas others focused on young adult vegetarians with very different characteristics). Third, some factors associated with BMD, such as the time that vegetarians and vegans had been following the diet, daily energy intake, number of hours engaged in physical activity, BMI, use of hormone replacement therapy, sunlight exposure, consumption of alcohol, and smoking behavior, could not be evaluated because this information was not reported for most of the studies. Another limitation is the reliance on self-reported measurements, which are prone to errors, for such data as BMI, physical activity, and fracture rates. The lack of dietary quality information for most studies could be considered another limitation. Furthermore, whether individuals had a low bone mass or osteoporosis prior to starting a vegetarian or vegan diet, which could influence the results, was not reported for any of the included studies. Moreover, for some of the studies, it was reported that the participants were mostly vegetarians or vegans or long-term vegetarians without specifying the duration of the diet, which make definitions sometimes ambiguous for interpretation. Finally, our study focused on the effect of plant-based diets on BMD. We grouped plant-based diets as vegetarians and vegans. Thus, behavioral omnivore diet patterns were against vegetarians and vegans. There was no definition of behavioral omnivore diet patterns in our included studies, which anecdotally lead to confusion for the readers.

## 5 Conclusions

The present meta-analysis indicated that plant-based diets may be correlated with lower BMD of women when compared with omnivore population. However, this does not diminish the fact that a plant-based diet can be a harmful option to the overall bone health of population and more prospective researches are needed to clear the impact of plant-based diets on bone health.

## Author contributions

**Conceptualization:** Huashan Pan.

**Data curation:** Mingjun Hu, Lijuan Zou.

**Formal analysis:** Huashan Pan, Shengcai He.

**Methodology:** Huashan Pan, Shengcai He.

**Software:** Huashan Pan, Shengcai He, Xingfa Ma, Haoyin Tan.

**Writing – original draft:** Huashan Pan, Shengcai He, Xingfa Ma, Haoyin Tan.

**Writing – review & editing:** Huashan Pan, Shengcai He, Xingfa Ma, Haoyin Tan.

## Supplementary Material

Supplemental Digital Content
