# Supplementary material for: The impact of plant-based diets on female bone mineral density: Evidence based on seventeen studies
Source: Medicine (Baltimore). 2021 Nov 19;100(46):e27480. doi: 10.1097/MD.0000000000027480 (PMC8601298; doi:10.1097/MD.0000000000027480)
Supplement: Supplemental Digital Content [file medi-100-e27480-s001.docx]

Table S1. Quality assessment of included studies.

| Included studies | AHRQ assessment criteria | | | | | | | | | | | Quality score |
| --- | --- | --- | --- | --- | --- | --- | --- | --- | --- | --- | --- | --- |
|  | 1 | 2 | 3 | 4 | 5 | 6 | 7 | 8 | 9 | 10 | 11 |  |
| Barr et al. 1998 | Yes | Yes | Unclear | Yes | Unclear | Yes | Yes | Yes | Yes | No | Unclear | 7 |
| Chiu et al. 1997 | Yes | Yes | Yes | Unclear | Unclear | Yes | Yes | Yes | Yes | Yes | No | 8 |
| Fontana et al. 2005 | Yes | Yes | Unclear | No | Unclear | Yes | Yes | Yes | Unclear | No | Yes | 6 |
| Ho-Pham et al. 2009 | Yes | Unclear | No | Yes | Unclear | Yes | No | Unclear | Unclear | No | Yes | 4 |
| Karavasiloglou et al. 2020 | Yes | Yes | Yes | Yes | No | Yes | No | Unclear | Unclear | No | Yes | 6 |
| Kim et al. 2007 | Yes | Yes | Yes | Yes | Unclear | Yes | No | Yes | Unclear | Yes | Yes | 8 |
| Knurick et al. 2015 | Yes | Yes | Yes | Yes | Yes | Yes | No | Yes | Unclear | No | Yes | 8 |
| Krivoskova et al. 2010 | Yes | Yes | Yes | Yes | Unclear | Yes | No | Yes | Unclear | No | Yes | 7 |
| Kaur et al. 2013 | Yes | Yes | No | Yes | Unclear | Yes | No | Yes | No | Yes | Unclear | 6 |
| Lloyd et al. 1991 | Yes | Yes | Unclear | Yes | Unclear | Yes | No | Yes | Unclear | No | Yes | 6 |
| Lau et al. 1998 | Yes | Yes | Yes | Yes | Unclear | Yes | Yes | Yes | Unclear | No | Yes | 8 |
| Outila et al. 2000 | Yes | Yes | No | Yes | Yes | Yes | No | Yes | Unclear | Unclear | Yes | 7 |
| Siani et al. 2003 | Yes | Yes | Yes | Yes | Unclear | Yes | No | Yes | Unclear | No | Unclear | 6 |
| Tesar et al. 1992 | Yes | Yes | Yes | Yes | Unclear | Yes | No | Yes | Unclear | No | Yes | 7 |
| Wang et al. 2008 | Yes | Yes | Unclear | Yes | Unclear | Yes | Yes | Yes | Yes | No | Yes | 8 |
| Xie et al. 2019 | Yes | Yes | Yes | Yes | Unclear | Yes | No | Yes | Unclear | Unclear | Yes | 7 |
| Ying-M et al. 2010 | Yes | Yes | Yes | Yes | Unclear | Yes | No | Yes | Unclear | Yes | Yes | 8 |

1) Define the source of information (survey, record review)

2) List inclusion and exclusion criteria for exposed and unexposed subjects (cases and controls) or refer to previous publications

3) Indicate time period used for identifying patients

4) Indicate whether or not subjects were consecutive if not population-based

5) Indicate if evaluators of subjective components of study were masked to other aspects of the status of the participants

6) Describe any assessments undertaken for quality assurance purposes (e.g., test/retest of primary outcome measurements)

7) Explain any patient exclusions from analysis

8) Describe how confounding was assessed and/or controlled.

9) If applicable, explain how missing data were handled in the analysis

10) Summarize patient response rates and completeness of data collection

11) Clarify what follow-up, if any, was expected and the percentage of patients for which incomplete data or follow-up was obtained
